# Fractal Gradation Effects on Dynamic Response and Failure of Cemented Coal Gangue Backfill Composites

**DOI:** 10.3390/ma19132784

**Published:** 2026-07-01

**Authors:** Yongjin Zhang, Hui Yang, Kangsheng Xue, Xin Qu, Cheng Li

**Affiliations:** 1 School of Architectural Engineering, Kaili University, Kaili 556011, China; asd18798012397@163.com (Y.Z.); yh7005@126.com (H.Y.); 2 State Key Laboratory of Intelligent Construction and Healthy Operation and Maintenance of Deep Earth Engineering, China University of Mining and Technology, Xuzhou 221116, China; ksxue@cumt.edu.cn; 3 School of Civil Engineering and Transportation, Anyang Institute of Technology, Anyang 455000, China

**Keywords:** cemented coal gangue backfill composite, fractal gradation, dynamic mechanical behavior, SHPB test, energy dissipation, failure mechanism

## Abstract

**Highlights:**

- Fractal gradation regulates dynamic response and impact failure.
- *D*_f_ = 2.41 gives the best overall bearing performance.
- Energy dissipation reflects gradation-induced impact damage.
- A skeleton–wave–energy–crack mechanism guides gradation design.

**Abstract:**

This study investigates the effect of coal gangue aggregate fractal gradation on the dynamic mechanical behavior and impact failure mechanism of cemented coal gangue backfill composites. Four aggregate gradations with different mass fractal dimensions were designed, and static compression and split Hopkinson pressure bar (SHPB) dynamic compression tests were conducted. The effects of fractal dimension and strain rate on stress–strain response, dynamic peak strength, dynamic increase factor (DIF), deformation modulus, energy dissipation, and failure morphology were analyzed. The results show that the composites exhibit a clear strain-rate strengthening effect, with dynamic strength, DIF, and deformation modulus increasing as strain rate increases. Aggregate fractal dimension has a nonlinear regulatory effect on mechanical performance. Among the four tested gradations, the specimen with *D*_f_ = 2.41 exhibits the best overall static and dynamic bearing performance, which is attributed to a more continuous coarse-particle skeleton and improved fine-particle filling. When the fractal dimension is too low, insufficient fine-particle filling leads to discontinuous contacts and larger pores; when it is too high, excessive fine particles weaken coarse-particle interlocking and promote matrix-dominated deformation. Energy analysis and failure observations further indicate that an intermediate fractal gradation improves energy absorption and delays unstable crack propagation. These findings provide a reference for gradation optimization and dynamic stability evaluation of coal gangue-based cemented backfill materials.

## 1. Introduction

With the large-scale exploitation of coal resources, the production of solid wastes such as coal gangue has continued to increase [1,2]. Long-term stockpiling of these wastes not only occupies substantial land resources but may also cause soil and water contamination, dust dispersion, and ecological degradation [3]. Using coal gangue as aggregate to prepare cemented backfill materials can realize the resource utilization of coal-based solid wastes and provide support for goaf filling, surrounding rock reinforcement, and ground pressure control [4,5]. Therefore, it has become an important approach for green mine construction and coordinated solid-waste disposal [6]. Cemented coal gangue backfill materials are composed of coal gangue aggregates, cementitious matrix, active fly ash components, and interfacial transition zones [7]. Their mechanical properties are jointly controlled by aggregate gradation, pore structure, cementitious matrix, and interparticle contact relationships [8]. In practical mining environments, backfill bodies are subjected not only to long-term ground pressure but also to dynamic disturbances such as blasting vibration, roof collapse impact, and mining-induced stress waves [9,10]. Therefore, clarifying the dynamic response and impact failure mechanism of cemented coal gangue backfill materials is essential for evaluating their impact stability and engineering service safety.

The split Hopkinson pressure bar (SHPB) test is widely used to investigate the dynamic mechanical behavior of rocks, concrete, and cement-based composites [11,12]. Previous studies have shown that rocks and rock-like materials generally exhibit a pronounced strain-rate effect under impact loading; their dynamic compressive strength, deformation modulus, and failure intensity usually increase with increasing strain rate, while the failure mode may evolve from blocky fracture to fragmentation or pulverization [13,14,15]. For cemented backfill materials, this dynamic response is further complicated by their multiphase and heterogeneous internal structure [16]. Aggregate distribution, pore defects, and interfacial transition zones can induce stress-wave reflection, transmission, and scattering, thereby affecting dynamic strength, deformation behavior, and damage evolution [17]. However, most existing studies on cemented backfill materials have focused on cement–tailings ratio, slurry concentration, curing age, water–binder ratio, or additives [18,19]. The quantitative role of aggregate gradation, especially its influence on the dynamic load-bearing response, remains insufficiently clarified.

Aggregate gradation is a key factor affecting the internal structure and load-bearing performance of cemented coal gangue backfill materials [20]. A reasonable aggregate gradation helps form a continuous and stable particle skeleton, while fine particles can fill the pores between coarse aggregates, improving material compactness and interfacial bonding [21]. In contrast, unreasonable gradation, such as discontinuous coarse-particle contact or an excessive proportion of fine particles, may increase pore defects, enhance stress concentration, and weaken the supporting effect of the particle skeleton [22]. Traditional gradation design mainly relies on empirical proportions or sieving curves, making it difficult to describe the heterogeneity and filling characteristics of particle systems using a unified structural parameter [23]. Fractal theory provides an effective method for quantitatively characterizing particle-size distribution [24]. By introducing the mass fractal dimension, complex gradation characteristics can be transformed into a structural parameter that links aggregate distribution with material properties [25,26]. Although previous studies have shown that fractal dimension can reflect gradation continuity, filling capacity, and compactness and can affect the static strength, deformation behavior, and long-term stability of cement-based materials [27,28], its role under dynamic impact loading has not been sufficiently examined.

Under dynamic loading, material failure is governed not only by its initial bearing capacity but also by the transmission, conversion, and dissipation of impact energy [29]. After a stress wave enters the specimen, part of the energy is reflected or transmitted, while the remaining energy is dissipated through crack initiation, interfacial friction, particle crushing, and matrix damage [30]. For cemented coal gangue backfill composites, different fractal gradations can alter particle skeleton continuity, pore distribution, and interfacial bonding, thereby changing stress-wave propagation paths, local stress concentration, and crack propagation modes [31]. An appropriate coarse–fine particle proportion may form a more stable interlocking skeleton and more tortuous crack paths, which is beneficial for improving dynamic bearing and energy dissipation capacities [32,33]. In contrast, excessively low or high fractal dimensions may promote interfacial debonding, pore expansion, or particle crushing, leading to premature impact failure [30,34]. Therefore, it is necessary to establish a clearer connection between fractal gradation structure and dynamic damage evolution by combining SHPB testing, energy analysis, and macro- and microscopic failure characterization.

Based on this motivation, this study uses the fractal gradation of coal gangue aggregates as a quantitative structural variable. Four aggregate gradation schemes with different mass fractal dimensions were designed, and cemented coal gangue backfill composite specimens were prepared. Static compression and SHPB dynamic compression tests were then conducted to evaluate the effects of fractal dimension and strain rate on stress–strain response, dynamic peak strength, dynamic increase factor, dynamic deformation modulus, and energy dissipation. In addition, macroscopic failure morphology and SEM observations were combined to analyze crack propagation, interfacial damage, and particle crushing characteristics. On this basis, a dynamic damage mechanism regulated by fractal gradation was proposed through the chain of “particle skeleton structure–stress-wave propagation–energy dissipation–crack propagation.” The findings provide a reference for gradation optimization, impact-resistance improvement, and dynamic stability evaluation of coal gangue-based cemented backfill materials.

## 2. Materials and Methods

### 2.1. Raw Materials

**Figure 1:**
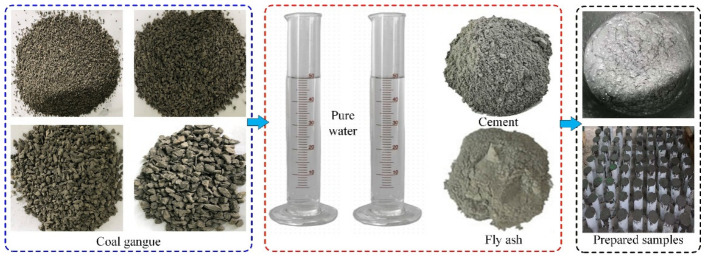
Raw materials and specimen preparation process.

The raw materials used in this study included coal gangue aggregate, ordinary Portland cement, fly ash, and mixing water, as shown in Figure 1. The coal gangue was collected from a mining area in Xinjiang, China. After natural drying, mechanical crushing, and graded sieving, aggregates with different particle-size ranges were obtained. The coal gangue particles were generally irregular in shape, with rough surfaces and distinct angular features, which are beneficial for enhancing mechanical interlocking between particles and providing skeleton support within the cemented system.

Ordinary Portland cement (P.O 42.5) was supplied by Xuzhou Qingshan Cement Manufacturing Co., Ltd., Xuzhou, China. The fly ash was obtained from a local coal-fired power plant in Xuzhou, China. The cement mainly provided early-stage hydration and cementation, forming a continuous cementitious matrix and providing the specimens with basic mechanical strength. Fly ash was used as a supplementary cementitious and filling material. Its fine particles can fill pores between aggregates and improve the compactness of the slurry. In addition, considering its relatively high SiO_2_ and Al_2_O_3_ contents, fly ash may contribute to later-age strength development through secondary reactions under alkaline conditions. However, because no XRD-based mineralogical analysis was conducted, the specific mineral phases and reaction products are not further discussed in this study. Tap water was used as the mixing water to satisfy the requirements of cement hydration and slurry workability.

Table 1 presents the major oxide compositions of cement, fly ash, and coal gangue. The coal gangue contains relatively high contents of SiO_2_ and Al_2_O_3_, indicating that it is a silica- and alumina-rich solid waste material. These oxide composition characteristics are consistent with its use as the main aggregate in cemented backfill composites. However, because XRD or equivalent mineralogical characterization was not conducted in this study, the specific mineral phases of coal gangue and fly ash are not identified or further interpreted here.

In the present study, the role of coal gangue aggregate is mainly discussed based on its measured oxide composition, particle-size distribution, and observed particle morphology. After crushing and sieving, the gangue particles exhibited irregular shapes, rough surfaces, and angular features, which are beneficial for forming mechanical interlocking and aggregate skeleton support within the cemented system. Therefore, the influence of coal gangue on the dynamic behavior of the composite is interpreted mainly from the perspectives of aggregate gradation, coarse–fine particle filling, pore structure, interfacial bonding, stress-wave propagation, and crack evolution, rather than from unsupported mineralogical phase assumptions.

Although XRD-based mineralogical characterization was not conducted in this study, the oxide compositions, particle-size characteristics, and particle morphology provide useful information for distinguishing the different functions of coal gangue and fly ash in the composite system. Coal gangue was used as the main aggregate, providing particle skeleton support and mechanical interlocking after crushing and sieving. In contrast, fly ash was used as a fine supplementary cementitious and filling material, contributing to pore filling and matrix compactness.

Therefore, although coal gangue and fly ash both contain relatively high proportions of SiO_2_ and Al_2_O_3_, they play different roles in the cemented coal gangue backfill composite and cannot be regarded as directly interchangeable in this study. Further mineralogical characterization, such as XRD analysis, would be useful for identifying the specific phase composition of the raw materials and reaction products, and this is acknowledged as a limitation of the present study.

### 2.2. Fractal Gradation Design and Specimen Preparation

To conduct dynamic mechanical tests on cemented coal gangue backfill composites, standard cylindrical specimens with dimensions of Φ50 mm × 50 mm were prepared according to the requirements of the split Hopkinson pressure bar (SHPB) test. The length-to-diameter ratio of the specimens was set to 1:1 to reduce end effects and size effects during stress wave propagation. Meanwhile, to minimize the influence of aggregate size on specimen homogeneity and test stability, the maximum particle size of coal gangue aggregate was controlled to be no greater than 10 mm, approximately 1/5 of the specimen diameter.

After crushing, the coal gangue was sieved using standard sieves with apertures of 1.0, 2.0, 4.0, 6.0, 8.0, and 10.0 mm, and the aggregate particle-size distribution was regulated based on mass fractal theory. The purpose of introducing the mass fractal model is to convert the discrete particle-size distribution of coal gangue aggregates into a single quantitative gradation parameter, namely the mass fractal dimension *D*_f_. A larger *D*_f_ corresponds to a higher proportion of fine particles, whereas a smaller *D*_f_ indicates a coarser aggregate system. To avoid symbol confusion, *d* is used to denote the particle size of coal gangue aggregate, and *D*_f_ is used to denote the mass fractal dimension of the aggregate particle-size distribution. For aggregates with particle sizes smaller than *d*, the cumulative particle number distribution function can be expressed as

(1) $$F(d)=\frac{N\left(d\right)}{N_{t}}$$

where *N*(*d*) is the number of aggregate particles with particle sizes smaller than *d*, and *N*_t_ is the total number of aggregate particles. According to fractal theory, the relationship between aggregate particle number and particle size can be expressed as

(2) $$N\left(d\right)=c_{0}+c_{1}{\left(\frac{d}{d_{\max}}\right)}^{-D_{f}}$$

where *D_f_* is the fractal dimension of the aggregate particle-size distribution, *d*_max_ is the maximum aggregate particle size, and *c*_0_ and *c*_1_ are fractal distribution parameters.

According to the boundary conditions *F*(*d*_max_) = 1 and *F*(*d*_min_) = 0, Equations (1) and (2) can be combined and rewritten as

(3) $$F(d)=\frac{d^{-D_{f}}-d_{\min}^{-D_{f}}}{d_{\max}^{-D_{f}}-d_{\min}^{-D_{f}}}$$

For aggregates with particle sizes smaller than *d*, the cumulative mass distribution function can be expressed as

(4) $$P(d)=\frac{M\left(d\right)}{M_{t}}$$

where *M*(*d*) is the cumulative mass of aggregates with particle sizes smaller than *d*, and *M*_t_ is the total mass of aggregates.

The aggregate mass is related to particle volume and particle number as follows:

(5) M=ρVN

(6) $$V\propto kd^{3}$$

where *ρ* is the aggregate density, *V* is the volume of a single aggregate particle, and *k* is the particle shape coefficient. By combining Equations (4)–(6) and differentiating, the following expression can be obtained:

(7) $$dP(d)=\frac{k\rho d^{3}}{Mt}dN$$

According to Equations (1), (3) and (7), the following equation can be derived:

(8) $$dN=N_{t}\frac{-D_{f}d^{-D_{f}-1}}{d_{\max}^{-D_{f}}-d_{\min}^{-D_{f}}}dd$$

Thus, the aggregate mass distribution function can be expressed as

(9) $$P(d)=\frac{-N_{t}k\rho D_{f}}{M_{t}(3-D_{f})(d_{\max}^{-D_{f}}-d_{\min}^{-D_{f}})}d^{3-D_{f}}+c$$

Based on the boundary conditions *P*(*d*_max_) = 1 and *P*(*d*_min_) = 0, the cumulative mass fraction of aggregates smaller than *P*_i_ can be obtained as

(10) $$P_{i}=\frac{d_{i}^{3-D_{f}}-d_{\min}^{3-D_{f}}}{d_{\max}^{3-D_{f}}-d_{\min}^{3-D_{f}}}$$

where *d*_i_ is the sieve aperture, and *P*_i_ is the cumulative mass fraction of aggregates smaller than *d*_i_. When $d_{\min}\ll d_{\max}$, $d_{\min}^{3-D_{f}}$ can be approximately neglected, and Equation (10) can be further transformed into

(11) $$\mathrm{lg}(\frac{M\left(d\right)}{M_{t}})\propto (3-D_{f})\mathrm{lg}(\frac{d}{d_{\max}})$$

**Figure 2:**
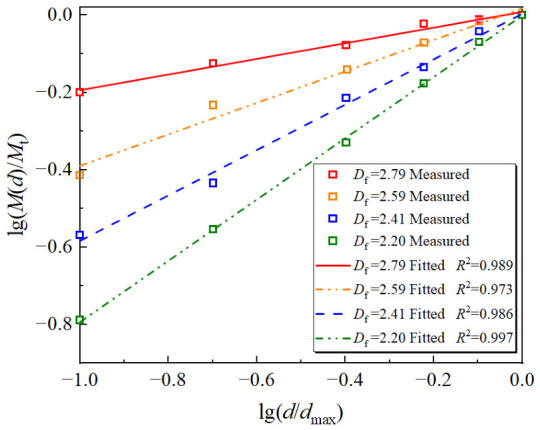
Particle-size distribution of coal gangue aggregates with different fractal dimensions.

Equation (11) indicates that $\mathrm{lg}(M\left(d\right)/M_{t})$ has an approximately linear relationship with $\mathrm{lg}(d/d_{\max})$, and the slope is $3-D_{f}$. Therefore, the fractal dimension of the aggregates can be determined from the cumulative mass distribution of different particle-size fractions. The particle-size distributions of coal gangue aggregates with different fractal dimensions are shown in Figure 2. As $D_{f}$ increases, the proportion of fine particles gradually increases, and the aggregate system changes from coarse-particle dominance to fine-particle filling dominance.

The selected fractal dimensions of 2.20, 2.41, 2.59, and 2.79 were designed to cover representative gradation states from coarse-particle-dominated to fine-particle-rich systems. Specifically, *D*_f_ = 2.20 represents a relatively coarse gradation with insufficient fine-particle filling; *D*_f_ = 2.41 and *D*_f_ = 2.59 represent intermediate gradations with improved coarse–fine particle coordination; and *D*_f_ = 2.79 represents a fine-particle-rich gradation in which the coarse-particle skeleton may be weakened. This range was determined by considering the available coal gangue particle-size fractions, practical backfill aggregate gradations, and preliminary mix-design considerations. Therefore, the four fractal dimensions were selected to examine how the transition from coarse skeleton dominance to fine-particle filling dominance affects the dynamic mechanical behavior of the cemented composite.

Specimens with different fractal dimensions were then prepared. First, cement and fly ash were thoroughly mixed, and mixing water was added to form a homogeneous slurry. Subsequently, coal gangue aggregates from different particle-size fractions were weighed according to the designed fractal gradation and mixed with the slurry to ensure that the cementitious slurry uniformly coated the aggregate particles. After initial setting, the specimens were demolded and cured under standard curing conditions at a temperature of 25 °C and a relative humidity of 95% for 28 days before subsequent testing.

To ensure comparability among different specimens, the mix proportion was kept constant for all groups, and only the coal gangue aggregate gradation was varied. The reported percentages refer to mass fractions of the total mixture. The mass fraction of coal gangue aggregate was 65%, cement was 16%, fly ash was 4%, and water was 15%. The binder refers to the total mass of cement and fly ash; therefore, the water-to-binder ratio was 0.75. After mixing, the mixture was placed in layers into Φ50 mm × 50 mm cylindrical molds and vibrated to remove entrapped air and improve specimen compactness. After initial setting, the specimens were demolded and cured in a standard curing chamber at 25 °C and 95% relative humidity for 28 days. After curing, the specimens were screened based on mass, dimensions, and wave velocity. Specimens with obvious external defects or large wave-velocity deviations were discarded, and specimens with good uniformity were selected for subsequent static and SHPB dynamic compression tests.

### 2.3. SHPB Dynamic Compression Test

**Figure 3:**
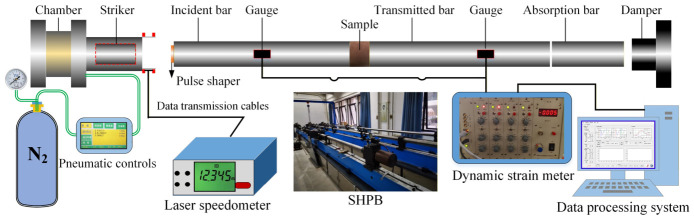
SHPB dynamic compression test system.

Dynamic compression tests of the cemented coal gangue backfill composites were conducted using a split Hopkinson pressure bar (SHPB) system, as shown in Figure 3. The system mainly consisted of a pneumatic driving device, striker bar, incident bar, transmitted bar, absorption bar, damping device, pulse shaper, strain acquisition system, laser velocimeter, and data processing system. The striker bar, incident bar, and transmitted bar were all made of 40 Cr high-strength steel, with a bar diameter of 50 mm, an elastic modulus of 208 GPa, a longitudinal wave velocity of approximately 5400 m/s, and a yield strength of approximately 800 MPa.

Before testing, both ends of each specimen were polished to ensure flatness and parallelism. To reduce friction between the specimen and the bar ends, a thin layer of molybdenum disulfide lubricant was uniformly applied to both specimen ends. During specimen installation, the specimen axis was aligned with the axes of the incident and transmitted bars to minimize the influence of eccentric loading. A pulse shaper was placed at the end of the incident bar to improve the incident waveform and extend the rise time of the stress wave, thereby facilitating stress equilibrium in the specimen during dynamic loading.

During the test, the striker bar was driven pneumatically to impact the incident bar, generating a compressive incident wave in the incident bar. When the incident wave reached the specimen interface, part of the wave was reflected at the incident end of the specimen to form a reflected wave, while the remaining part propagated through the specimen into the transmitted bar to form a transmitted wave. The incident, reflected, and transmitted strain signals were collected using strain gauges attached to the incident and transmitted bars and recorded using a dynamic strain meter and data acquisition system. The velocity of the striker bar was measured using a laser velocimeter.

Based on one-dimensional stress wave propagation theory, when stress equilibrium was achieved at both ends of the specimen, the dynamic stress, strain rate, and dynamic strain of the specimen were calculated using the three-wave method:

(12) $$\left\{\begin{array}{l} \begin{array}{l} P_{1}{=A}_{r}E_{0}(\varepsilon_{i}(t)+\varepsilon_{r}(t))\\ P_{2}=A_{r}E_{0}\varepsilon_{t}(t) \end{array}\\ \dot{\varepsilon }=\frac{C_{s}}{L_{s}}(\varepsilon_{i}(t)-\varepsilon_{r}(t)-\varepsilon_{t}(t))\\ \varepsilon =\frac{C_{s}}{L_{s}}\int_{0}^{t}(\varepsilon_{i}(t)-\varepsilon_{r}(t)-\varepsilon_{t}(t))dt \end{array}\right.$$

where *A*_r_ is the ratio of the cross-sectional area of the bar to that of the specimen; *E*_0_, *C*_s_ and *L*_s_ are the elastic modulus of the bar, the wave velocity in the bar, and the specimen length, respectively; $\varepsilon_{i}(t)$, $\varepsilon_{r}(t)$ and $\varepsilon_{t}(t)$ represent the incident, reflected, and transmitted strains, respectively.

To investigate the coupled effects of fractal dimension and strain rate on the dynamic mechanical behavior of the material, four groups of specimens with different fractal dimensions were tested under four impact velocity levels of 4, 5, 6, and 7 m/s. These different impact velocities corresponded to different strain-rate levels, enabling dynamic compression tests under multiple strain-rate conditions. Considering the heterogeneity of cemented backfill composites and the scatter of dynamic test results, five repeated tests were conducted for each test condition, and additional backup specimens were prepared to ensure the reliability and completeness of the experimental data.

### 2.4. Stress Equilibrium Verification and Data Validity

In SHPB dynamic compression tests, stress equilibrium at both ends of the specimen is a fundamental prerequisite for ensuring the validity of the experimental data. If the specimen does not reach stress equilibrium before failure, the dynamic stress–strain relationship obtained based on one-dimensional stress wave theory may contain significant errors. Therefore, in this study, the incident, reflected, and transmitted waves during the loading process of a typical specimen were analyzed, and the superposition of the incident and reflected waves was compared with the transmitted wave to verify the stress equilibrium state of the specimen during dynamic loading.

**Figure 4:**
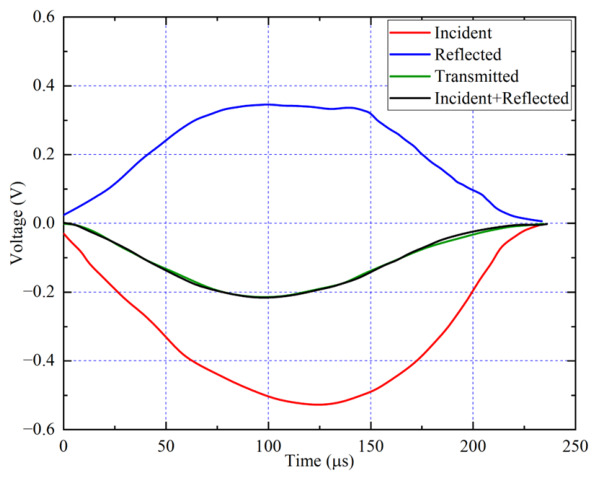
Stress equilibrium verification.

As shown in Figure 4, during a typical dynamic loading process, the superposed curve of the incident and reflected waves is generally consistent with the transmitted wave curve in terms of variation trend, indicating that the stress states at the two ends of the specimen are approximately identical. This demonstrates that the specimen can satisfy the stress equilibrium requirement during the main loading stage. The result confirms that the dynamic stress–strain curves and related mechanical parameters obtained in this study are reliable and can be used for the subsequent analysis of dynamic strength, dynamic deformation modulus, DIF, and energy dissipation.

It should be noted that Figure 4 presents a representative example of stress equilibrium verification. In this study, the incident, reflected, and transmitted wave signals of all valid SHPB tests were checked during data processing. Only the specimens that satisfied approximate stress equilibrium during the main loading stage were used for calculating the dynamic stress–strain curves and related mechanical parameters. Tests with abnormal waveforms, obvious eccentric loading, premature end crushing, or poor signal quality were excluded from the valid dataset. This procedure was adopted to reduce the influence of specimen heterogeneity and testing uncertainty on the dynamic parameters.

In addition, the strain rate reported in this study refers to the measured strain rate calculated from the SHPB signals rather than the nominal impact velocity. Although the impact velocities were set to 4, 5, 6, and 7 m/s for all gradation groups, the measured strain-rate ranges differ slightly among specimens because of differences in specimen stiffness, wave impedance, damage evolution, and failure duration. Therefore, comparisons among different fractal-dimension groups were made based on the measured strain rate and the fitted relationships between mechanical parameters and strain rate, rather than by assuming identical strain rates under the same nominal impact velocity.

## 3. Results and Discussion

### 3.1. Static Mechanical Properties

**Figure 5:**
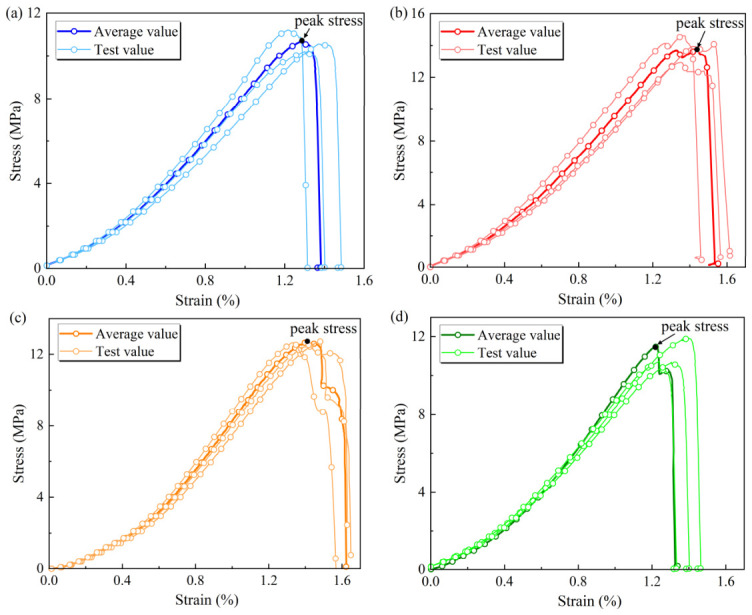
Uniaxial compressive stress–strain curves of cemented coal gangue backfill composites with different aggregate fractal dimensions: (**a**) *D*_f_ = 2.20; (**b**) *D*_f_ = 2.41; (**c**) *D*_f_ = 2.59; and (**d**) *D*_f_ = 2.79.

**Figure 6:**
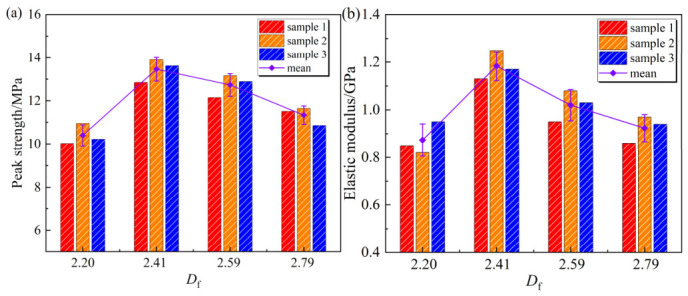
Static mechanical parameters of cemented coal gangue backfill composites with different aggregate fractal dimensions: (**a**) peak strength; (**b**) elastic modulus.

To clarify the basic mechanical properties of the cemented coal gangue backfill composites with different aggregate fractal dimensions, uniaxial compression tests were conducted on specimens with different fractal gradations. The corresponding stress–strain curves are shown in Figure 5. In Figure 5, the stress–strain responses are presented separately for the four fractal-dimension groups, namely *D*_f_ = 2.20, 2.41, 2.59, and 2.79, to more clearly show the deformation characteristics of each gradation condition. The peak strength and elastic modulus obtained from the valid specimens are further summarized in Figure 6.

As shown in Figure 5, the uniaxial compressive stress–strain curves of specimens with different fractal dimensions exhibit generally similar shapes and typical quasi-brittle characteristics. The curves can be divided into four stages: initial compaction, approximately linear elasticity, pre-peak nonlinear deformation and progressive damage, and post-peak failure. When the stress approaches the peak value, the curve slope gradually decreases, indicating the initiation and progressive development of microstructural damage, including local interfacial debonding, matrix cracking, particle rearrangement, frictional sliding at weak interfaces, and microcrack initiation. It should be emphasized that this pre-peak nonlinear deformation does not represent classical plastic hardening. Cemented coal gangue backfill composites are heterogeneous quasi-brittle materials composed of coal gangue aggregates, cementitious matrix, pores, and interfacial transition zones. Therefore, the observed nonlinearity is mainly caused by progressive pore closure, skeleton adjustment, interfacial damage, local particle rearrangement, and damage accumulation before peak stress, rather than by true plasticity in the conventional sense. After the peak stress is reached, the specimens enter the post-peak failure stage, and the load-bearing capacity decreases rapidly due to crack coalescence and structural instability.

The peak strength, deformation stiffness, and post-peak response differ markedly among specimens with different fractal dimensions, indicating that aggregate fractal gradation significantly affects the internal structure and load-bearing capacity of the specimens. When the fractal dimension is low, the proportion of coarse particles is relatively high, particle contacts are discontinuous, and pores and interfacial defects are more developed. These features lead to pronounced local stress concentration during loading and thus reduce the overall bearing capacity. At an intermediate fractal dimension, the coarse and fine particles are more reasonably proportioned. Fine particles can effectively fill the pores between the coarse-particle skeleton, forming a more continuous load-bearing skeleton and a denser cemented structure; consequently, the specimens exhibit higher strength and stiffness. When the fractal dimension further increases, the proportion of fine particles becomes excessive, the direct contact and interlocking effect among coarse particles are weakened, and the material relies more heavily on the cementitious matrix and fine-particle-rich regions for load bearing, resulting in a decline in overall mechanical performance.

It should be noted that some high-fractal-dimension specimens may exhibit a relatively extended deformation process or a larger failure strain in individual stress–strain curves. This phenomenon does not necessarily indicate an improvement in load-bearing capacity. For the *D*_f_ = 2.79 group, excessive fine particles may promote progressive compaction, particle rearrangement, and local crushing in fine-particle-rich regions during loading. These processes can delay complete macroscopic failure to some extent and lead to a longer deformation process. However, the continuous coarse-particle skeleton is weakened, and the effective stress-transfer path becomes less stable. Therefore, the evaluation of static mechanical performance should be based mainly on peak strength and elastic modulus rather than only on the failure strain of an individual curve.

Figure 6 further shows the variations in peak strength and elastic modulus with aggregate fractal dimension. The uniaxial compressive strength exhibits a clear nonmonotonic trend with increasing fractal dimension. As the fractal dimension increases from *D*_f_ = 2.20 to *D*_f_ = 2.41, the peak strength increases from approximately 10.40 MPa to 13.49 MPa. When the fractal dimension further increases to *D*_f_ = 2.59 and *D*_f_ = 2.79, the peak strength decreases to approximately 12.72 MPa and 11.21 MPa, respectively, showing an overall increase–decrease trend. The elastic modulus follows a similar trend to the peak strength and reaches its maximum value of approximately 1.18 GPa at *D*_f_ = 2.41.

The variation in elastic modulus is not controlled only by the pore-filling effect of fine particles, but also by the stiffness and continuity of the load-transfer skeleton. At a low fractal dimension, the aggregate system is dominated by coarse particles. Although coarse particles have relatively high stiffness, insufficient fine-particle filling leads to discontinuous interparticle contacts, large pores, and weak interfacial transition zones. Under this condition, the applied load cannot be transferred through a stable and continuous particle skeleton, resulting in a relatively low elastic modulus. When the fractal dimension increases to an intermediate level, fine particles effectively fill the voids between coarse particles, improve particle packing density, and enhance the contact continuity between aggregates and the cementitious matrix. The coarse particles still provide the main skeleton support, while the fine particles improve contact stiffness and reduce local deformation. Therefore, the elastic modulus increases.

However, when the fractal dimension becomes too high, the excessive fine-particle content weakens the direct contact and mechanical interlocking among coarse particles. Under a fixed binder content, a larger amount of fine particles also increases the total specific surface area of the aggregate system, which may reduce the effective coating quality of the cementitious matrix and promote fine-particle agglomeration or weak fine-particle-rich zones. As a result, the load-transfer path becomes more dependent on the cementitious matrix and fine-particle-dominated regions rather than on a continuous coarse-particle skeleton. These regions have lower effective skeleton stiffness and are more prone to local compaction and interfacial deformation. Consequently, further increasing the fine-particle proportion can reduce the elastic modulus, even though the pore-filling effect is enhanced.

Considering that only four fractal-dimension levels were tested, the relationship between fractal dimension and static mechanical parameters was interpreted mainly from the measured values and the observed nonmonotonic trend rather than from an overextended empirical regression. As shown in Figure 6, both peak strength and elastic modulus increase first and then decrease with increasing fractal dimension. Among the four tested gradation conditions, the *D*_f_ = 2.41 specimens exhibit the highest peak strength and elastic modulus, indicating the best overall static mechanical performance within the tested range. Therefore, the term “optimal” in the revised manuscript refers only to the best-performing gradation among the four tested conditions, rather than a universal optimum fractal dimension for all cemented coal gangue backfill composites.

These results indicate that the aggregate fractal dimension significantly regulates the static bearing capacity of cemented coal gangue backfill composites. Among the four tested gradation conditions, the gradation structure corresponding to *D*_f_ = 2.41 provides better particle filling and skeleton continuity, enabling the specimens to achieve higher structural compactness, stronger stress-transfer capacity, and greater deformation resistance. In contrast, either insufficient fine-particle filling at a low fractal dimension or excessive fine-particle content at a high fractal dimension can reduce the effective load-transfer capacity of the composite. Because the static mechanical properties reflect the initial structural state of the specimens, these differences can further influence stress-wave propagation, dynamic strength enhancement, and energy dissipation under impact loading. Therefore, in the subsequent dynamic analysis, the static strength is used as a reference, and the dynamic increase factor is introduced to further investigate the coupled effects of fractal dimension and strain rate on the dynamic mechanical behavior of the material.

### 3.2. Dynamic Mechanical Properties

#### 3.2.1. Dynamic Stress–Strain Response

Before analyzing the dynamic mechanical parameters, it should be noted that five repeated SHPB tests were conducted for each nominal test condition to reduce the influence of material heterogeneity. During data processing, specimens with abnormal waveforms, obvious eccentric loading, premature end crushing, or poor stress equilibrium were excluded from the valid dataset. The curves and mechanical parameters reported in this section were obtained from valid tests and are used to reveal the main effects of strain rate and fractal gradation. Because the measured strain rates and failure processes varied slightly among repeated SHPB tests, the discussion focuses on robust trends with strain rate and fractal dimension rather than on isolated data points. A full statistical uncertainty analysis for all dynamic parameters was not included in the present study, which is acknowledged as a limitation and should be improved in future work.

**Figure 7:**
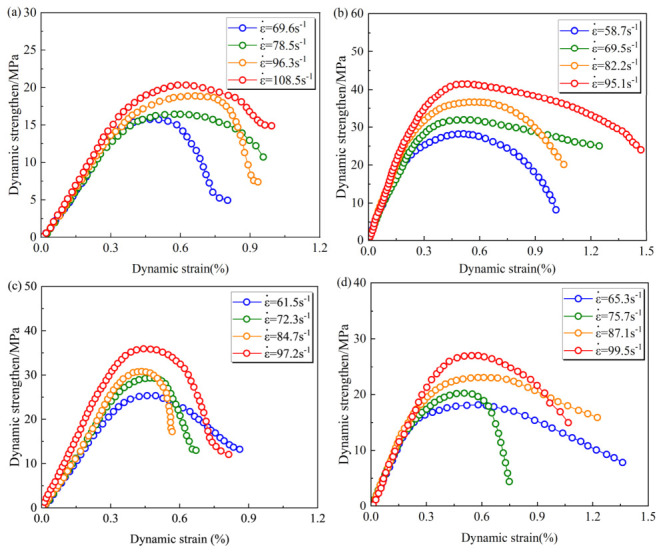
Dynamic stress–strain curves of specimens with different aggregate fractal dimensions: (**a**) *D*_f_ = 2.20; (**b**) *D*_f_ = 2.41; (**c**) *D*_f_ = 2.59; and (**d**) *D*_f_ = 2.79.

The dynamic stress–strain curves of specimens with different aggregate fractal dimensions under different strain-rate conditions are shown in Figure 7. Overall, both strain rate and aggregate fractal dimension have significant effects on the dynamic mechanical response of the specimens. Compared with the static uniaxial compression curves, the dynamic stress–strain curves exhibit a steeper overall slope, a markedly higher peak stress, and a slightly increased peak strain, indicating that the cemented coal gangue backfill composites exhibit a typical strain-rate strengthening effect.

Under impact loading, the initial compaction stage of most specimens is relatively short, and some curves enter an approximately linear growth stage at the early loading stage. This indicates that under high strain-rate loading, the stress wave is transmitted into the specimen within a very short time, and the particle skeleton and cementitious matrix begin to jointly bear the impact load before the primary pores and microcracks are fully closed. As the strain rate increases, the peaks of the dynamic stress–strain curves shift upward, and the peak stress continues to increase. For example, for the specimen with *D*_f_ = 2.20, when the strain rate increases from approximately 69.6 s^−1^ to 96.3 s^−1^, the dynamic peak strength increases from approximately 15.5 MPa to 19.9 MPa. This indicates that increasing the loading rate can significantly enhance the instantaneous load-bearing capacity of the specimen.

The strain-rate strengthening effect and the abrupt post-peak failure at high strain rates are not contradictory, but correspond to different stages of the dynamic loading process. Before the peak stress is reached, the loading duration under high strain-rate conditions is extremely short. Primary defects, interfacial cracks, and matrix microcracks do not have sufficient time to undergo stable propagation and gradual coalescence. Meanwhile, the inertia effect and transient confinement associated with rapid loading restrict lateral deformation and delay effective crack opening. Consequently, the specimen can withstand a higher instantaneous stress, resulting in the observed strain-rate strengthening effect. However, when the accumulated damage and stored elastic strain energy reach a critical level near the peak stress, the failure process becomes highly unstable. Multiple microcracks may nucleate, propagate, branch, and coalesce almost simultaneously within a very short time. The rapid release of impact energy then causes damage localization, sudden loss of load-bearing capacity, and severe fragmentation. Therefore, high strain rate delays stable crack growth before the peak, but promotes rapid unstable crack coalescence and abrupt failure after the peak. This two-stage mechanism explains why the specimens exhibit both increased dynamic strength and more sudden post-peak failure under high strain-rate loading.

Because the measured strain rates vary slightly among different fractal-dimension groups, the comparison of dynamic stress–strain responses was conducted using the measured strain-rate values. When comparing specimens with different fractal dimensions, emphasis was placed on curves at comparable strain-rate levels and on the fitted trends of dynamic mechanical parameters with strain rate. From the perspective of fractal dimension, at comparable strain-rate levels, the specimen with *D*_f_ = 2.41 generally exhibits the highest peak stress, and the slope of the quasi-elastic stage is also relatively large, indicating that this gradation condition provides higher dynamic stiffness and load-bearing capacity. The specimen with *D*_f_ = 2.59 ranks second, whereas the specimens with *D*_f_ = 2.20 and *D*_f_ = 2.79 exhibit relatively lower dynamic peak strengths. Overall, the dynamic strength first increases and then decreases with increasing fractal dimension, which is consistent with the trend observed in the static tests. This demonstrates that the coarse–fine particle synergistic structure corresponding to an intermediate fractal dimension is favorable for forming a continuous and stable load-bearing skeleton, improving stress-wave transmission continuity, and reducing local stress concentration.

In addition, the fractal dimension also affects the post-peak response characteristics of the specimens. The low-fractal-dimension specimens exhibit a rapid post-peak stress drop, indicating that when the proportion of coarse particles is high, the skeleton continuity is insufficient, and once cracks propagate through weak interfaces, sudden instability is likely to occur. In contrast, the specimens with *D*_f_ = 2.41 and *D*_f_ = 2.59 still maintain a certain residual load-bearing capacity after the peak, indicating better internal structural integrity and a stronger ability to absorb and dissipate energy during failure.

#### 3.2.2. Evolution Characteristics of Dynamic Peak Strength

**Figure 8:**
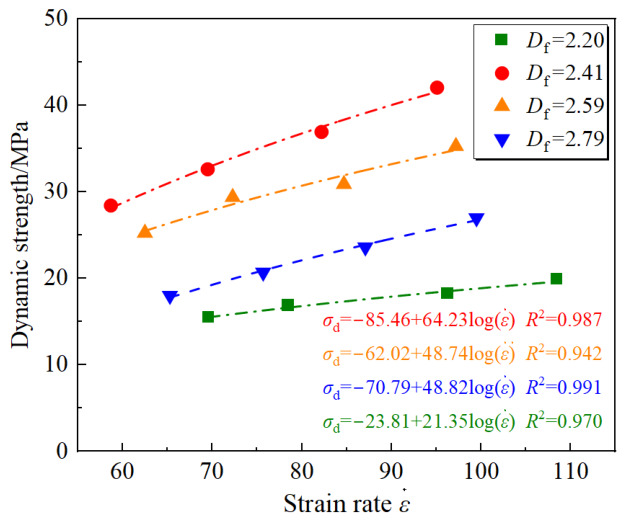
Relationship between dynamic peak strength and strain rate for specimens with different aggregate fractal dimensions.

To further analyze the strain-rate effect of cemented coal gangue backfill composites with different aggregate fractal dimensions, the relationship between dynamic peak strength and strain rate was fitted, as shown in Figure 8. The dynamic peak strength of all four groups of specimens increases with increasing strain rate, and this relationship can be described by a logarithmic function:

(13) $$\sigma_{d}=m\log(\dot{\varepsilon })+f$$

where *σ*_d_ is the dynamic peak strength, MPa; $\dot{\varepsilon }$ is the strain rate, s^−1^; and *m* and *f* are fitting parameters.

As shown in Figure 8, the fitting correlation coefficients of the specimens with different fractal dimensions are relatively high. When the fractal dimensions are *D*_f_= 2.20, 2.41, 2.59 and 2.79, the coefficients of determination *R*^2^ are 0.970, 0.987, 0.942, and 0.991, respectively, indicating a good logarithmic correlation between dynamic peak strength and strain rate.

Comparing the specimens with different fractal dimensions, the specimen with *D*_f_ = 2.41 exhibits the highest dynamic peak strength at all strain-rate levels, increasing from approximately 28.86 MPa to 42.28 MPa. The specimen with *D*_f_ = 2.59 ranks second, with its dynamic peak strength increasing from approximately 25.29 MPa to 36.96 MPa. The dynamic peak strength of the specimen with *D*_f_ = 2.79 increases from approximately 18.2 MPa to 28.4 MPa, whereas the specimen with *D*_f_ = 2.20 shows the lowest dynamic peak strength, increasing only from approximately 15.49 MPa to 19.86 MPa. These results further indicate that the gradation structure corresponding to an intermediate fractal dimension still provides superior load-bearing performance under dynamic loading.

The fitting parameter can be used to characterize the sensitivity of dynamic peak strength to strain rate. According to the fitting results, the specimen with *D*_f_ = 2.41 has the largest *m* value of 64.23, indicating that its dynamic strength is the most sensitive to strain-rate variation and that it experiences the greatest strength enhancement under impact loading. The *m* values of the specimens with *D*_f_ = 2.59 and *D*_f_ = 2.79 are 48.74 and 48.82, respectively, indicating similar strain-rate sensitivities. In contrast, the specimen with *D*_f_ = 2.20 has the lowest *m* value of 21.35, suggesting a relatively weak dynamic strengthening effect.

The above differences are mainly attributed to the fact that different fractal gradations alter the internal particle skeleton, pore distribution, and interfacial bonding state of the specimens. When the fractal dimension is within a reasonable range, coarse particles form a stable skeleton while fine particles fill the skeleton pores, thereby improving stress-wave transmission continuity and overall load-bearing capacity. When the fractal dimension is too low, the proportion of coarse particles is excessive, particle contacts are more discrete, and more pores and interfacial defects are present. Under impact loading, cracks tend to propagate rapidly along weak interfaces, thereby limiting the increase in dynamic strength.

To quantitatively characterize the increase in specimen strength under impact loading relative to static strength, the dynamic increase factor (DIF) is introduced as follows:

(14) $$DIF=\frac{\sigma_{d}}{\sigma_{s}}$$

where *σ*_d_ is the dynamic peak strength under a given strain rate, and *σ*_s_ is the static uniaxial compressive strength of the specimen with the corresponding fractal dimension.

**Figure 9:**
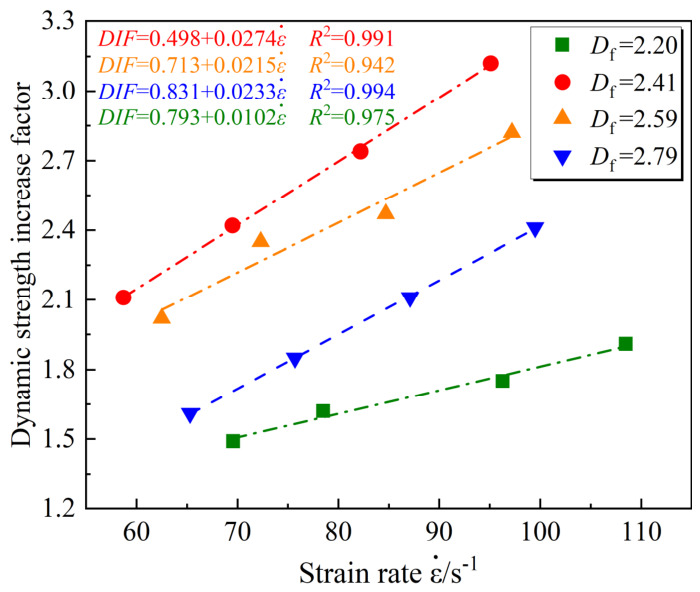
Variation in DIF with strain rate for specimens with different aggregate fractal dimensions.

Figure 9 shows that the DIF values of all four groups of specimens continuously increase with increasing strain rate, indicating that the cemented coal gangue backfill composites exhibit a pronounced dynamic strengthening effect. Within the strain-rate range tested in this study, the DIF values of all specimen groups show a clear increasing trend with strain rate, suggesting that the dynamic strength enhancement of the material is relatively stable.

From the perspective of fractal dimension, DIF first increases and then decreases with increasing fractal dimension. Among the specimens, the *D*_f_ = 2.41 group exhibits the highest DIF values under all strain-rate conditions. As the strain rate increases from approximately 58.7 s^−1^ to 95.1 s^−1^, its DIF increases from approximately 2.11 to 3.12, indicating the most significant dynamic strengthening effect. In contrast, the specimen with *D*_f_ = 2.20 exhibits the lowest DIF, increasing only from 1.48 to 1.90. This result indicates that specimens with an intermediate fractal dimension not only possess higher static strength but also exhibit a stronger strength enhancement capacity under impact loading.

#### 3.2.3. Evolution Characteristics of Dynamic Deformation Modulus

**Figure 10:**
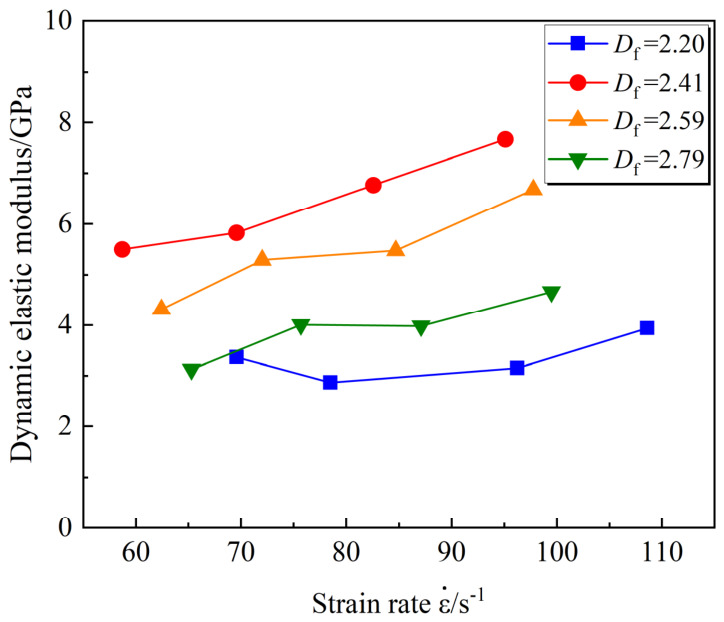
Variation in dynamic deformation modulus with strain rate for specimens with different fractal dimensions.

To further characterize the deformation resistance of cemented coal gangue backfill composites under impact loading, the dynamic deformation modulus was calculated as the ratio of the dynamic peak stress to the corresponding peak strain. The variation in dynamic deformation modulus is shown in Figure 10.

As shown in Figure 10, the dynamic deformation modulus generally increases with increasing strain rate, indicating that the specimens exhibit stronger instantaneous deformation resistance under higher strain-rate conditions. Taking the specimen with *D*_f_ = 2.41 as an example, when the strain rate increases from 58.7 s^−1^ to 95.1 s^−1^, the dynamic deformation modulus increases from approximately 5.48 GPa to 7.65 GPa, corresponding to an increase of approximately 39.6%. For the specimen with *D*_f_ = 2.20, the dynamic deformation modulus increases from 3.35 GPa to 3.92 GPa, with an increase of approximately 17.0%. These results indicate that increasing the loading rate not only enhances the dynamic strength of the material but also significantly improves its overall stiffness under impact loading.

The dynamic deformation modulus varies markedly among specimens with different fractal dimensions. With increasing fractal dimension, the dynamic deformation modulus generally first increases and then decreases. Among all groups, the specimen with *D*_f_ = 2.41 consistently exhibits the highest dynamic deformation modulus at all strain-rate levels, indicating that this gradation condition provides the best dynamic deformation resistance. This can be attributed to the more reasonable combination of coarse and fine particles at an intermediate fractal dimension. Under this condition, coarse particles form a stable load-bearing skeleton, while fine particles effectively fill pores and improve the interfacial transition zone, enabling the aggregates and cementitious matrix to cooperatively resist impact-induced deformation.

When the fractal dimension is relatively low, the proportion of coarse particles is high, particle contacts are discontinuous, and more pores and interfacial defects are present. These features make local deformation more likely under impact loading, resulting in a lower dynamic deformation modulus. When the fractal dimension is too high, the proportion of fine particles increases excessively. Although this can further enhance local pore filling, it weakens the direct contact and interlocking among coarse particles and reduces the continuity of the load-bearing skeleton. Under a fixed binder content, the increased specific surface area of fine particles may also reduce the effective coating and bonding quality of the cementitious matrix, resulting in weak fine-particle-rich zones. Consequently, the specimen becomes more dependent on matrix-dominated deformation rather than skeleton-dominated load transfer, which limits the dynamic deformation modulus.

### 3.3. Dynamic Energy Evolution

The failure process of rocks and cemented materials under impact loading is essentially a process in which external input energy is continuously transmitted, transformed, and dissipated within the material. In SHPB dynamic compression tests, the energy components mainly include incident energy, reflected energy, transmitted energy, and dissipated energy. The incident energy represents the total energy input into the system by the striker impact; the reflected energy reflects the ability of the specimen to reflect the incident stress wave; the transmitted energy represents the energy that continues to propagate after the stress wave passes through the specimen; and the dissipated energy is mainly consumed by microcrack initiation, propagation, coalescence, interfacial friction, and particle crushing inside the specimen. Therefore, the dissipated energy can effectively reflect the damage development and energy absorption capacity of the material during impact failure.

Based on one-dimensional stress wave theory, the energy components can be calculated from the incident, reflected, and transmitted waves as follows:

(15) $$\left\{\begin{array}{l} E_{\mathrm{in}}=E_{0}C_{s}A_{s}\int_{0}^{t}{\varepsilon_{i}}^{2}(t)dt\\ E_{\mathrm{re}}=E_{0}C_{s}A_{s}\int_{0}^{t}{\varepsilon_{r}}^{2}(t)dt\\ E_{\mathrm{tr}}=E_{0}C_{s}A_{s}\int_{0}^{t}\varepsilon_{t}^{2}(t)dt\\ E_{\mathrm{di}}=E_{\mathrm{in}}-(E_{\mathrm{re}}+E_{\mathrm{tr}}) \end{array}\right.$$

where *A*_b_ is the cross-sectional area of the bar, *C*_s_ is the stress wave velocity in the bar, *E*_0_ is the elastic modulus of the bar, and *ε*_i_(t), *ε*_r_(t) and *ε*_t_(t) are the incident, reflected, and transmitted strain signals, respectively. To eliminate the influence of specimen size on the energy dissipation results, the volumetric dissipated energy was used to characterize the energy absorption capacity of the material:

(16) $$\eta_{d}=\frac{E_{\mathrm{di}}}{V_{s}}$$

where *η*_d_ is the volumetric dissipated energy, and *V*_s_ is the specimen volume.

**Figure 11:**
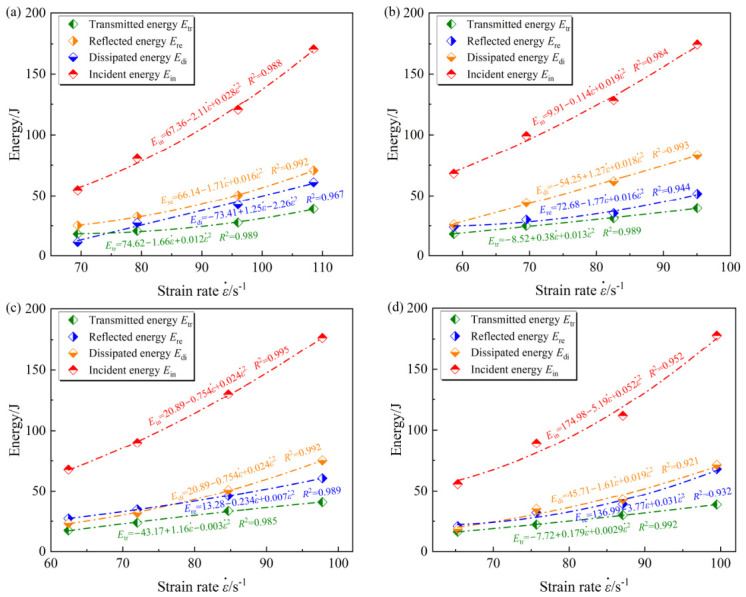
Variation in energy components with strain rate for specimens with different aggregate fractal dimensions: (**a**) *D*_f_ = 2.20; (**b**) *D*_f_ = 2.41; (**c**) *D*_f_ = 2.59; and (**d**) *D*_f_ = 2.79.

Figure 11 shows the variation in different energy components with strain rate for specimens with different fractal dimensions. As shown in Figure 11, the incident energy, reflected energy, transmitted energy, and dissipated energy of all specimen groups increase continuously with increasing strain rate. Among them, the incident energy shows the largest increase, indicating that the input energy of the system increases significantly as the impact velocity increases. Meanwhile, the dissipated energy and transmitted energy also increase, suggesting that more external energy is consumed in internal damage evolution and stress wave propagation.

Taking the specimen with *D*_f_ = 2.41 as an example, as the strain rate increases from approximately 58.7 s^−1^ to 95.1 s^−1^, the incident energy increases from approximately 68.5 J to 174.7 J, while the dissipated energy increases from approximately 25.6 J to 82.7 J. The increase in dissipated energy is more pronounced than that of the reflected and transmitted energies, indicating that this gradation condition enables stronger energy absorption and damage dissipation under high-strain-rate impact loading.

For the same fractal dimension, the incident, reflected, transmitted, and dissipated energies all increase with increasing strain rate; however, different energy components show different sensitivities to fractal dimension. The incident energy is mainly controlled by impact velocity, and therefore its variation among specimens with different fractal dimensions is relatively limited. In contrast, the transmitted energy and dissipated energy are more sensitive to aggregate fractal dimension and can more directly reflect the regulatory effect of the internal structure on stress wave propagation and damage evolution.

In terms of transmitted energy, at similar strain-rate levels, the specimens with *D*_f_ = 2.41 and *D*_f_ = 2.59 generally exhibit higher transmitted energy than those with *D*_f_ = 2.20 and *D*_f_ = 2.79, indicating that stress waves can pass more effectively through specimens with intermediate fractal dimensions. This is mainly because the coarse and fine particles are more reasonably distributed under these gradation conditions, the aggregate skeleton is more continuous, and pores and interfacial defects are relatively fewer. As a result, scattering and reflection losses during stress wave propagation are reduced, allowing more energy to pass through the specimen as transmitted energy.

The dissipated energy also varies markedly with fractal dimension. The specimens with *D*_f_ = 2.41 and *D*_f_ = 2.59 show more significant increases in dissipated energy at medium and high strain rates, indicating that specimens with intermediate fractal dimensions can absorb and dissipate more energy under impact loading. Therefore, specimens with intermediate fractal dimensions exhibit both higher transmitted energy and higher dissipated energy, suggesting that their internal structure is favorable for stable stress wave propagation and can also absorb more impact energy during failure. This indicates superior dynamic bearing capacity and impact resistance.

**Figure 12:**
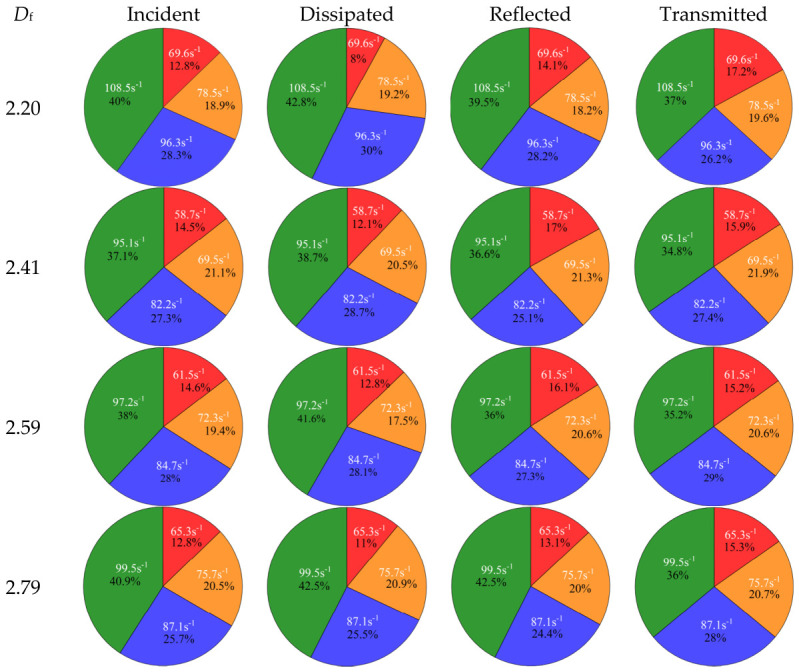
Energy distribution proportions of cemented coal gangue backfill composites under representative fractal-dimension and strain-rate conditions.

**Figure 13:**
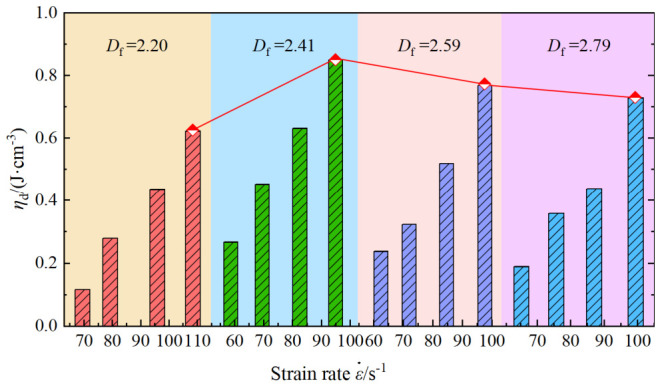
Variation in volumetric dissipated energy with strain rate for specimens with different fractal dimensions.

Figure 12 further illustrates the relative proportions of reflected, transmitted, and dissipated energies under representative fractal-dimension and strain-rate conditions. The pie charts are used to provide a visual comparison of the energy-partitioning characteristics, whereas the quantitative evolution of energy components is mainly evaluated from Figure 11 and the volumetric dissipated energy shown in Figure 13. Overall, the energy-distribution results indicate that impact input energy is primarily governed by strain rate, while the proportion and magnitude of dissipated energy are also affected by the internal gradation structure. Specimens with intermediate fractal dimensions tend to consume more energy through crack initiation, interfacial friction, matrix damage, and local particle crushing before failure.

Figure 13 shows the variation in volumetric dissipated energy with strain rate for specimens with different fractal dimensions. The volumetric dissipated energy *η*_d_ increases significantly with increasing strain rate, indicating that as the impact velocity increases, more energy per unit volume is consumed by crack propagation, interfacial friction, and particle crushing, and the degree of dynamic damage increases accordingly.

Among all specimen groups, the specimen with *D*_f_ = 2.41 maintains a relatively high *η*_d_ at all strain-rate levels, increasing from approximately 0.27 J/cm^3^ to 0.85 J/cm^3^, corresponding to an increase of approximately 214.8%. From the perspective of fractal dimension, the volumetric dissipated energy generally first increases and then decreases with increasing fractal dimension, and specimens with intermediate fractal dimensions exhibit stronger impact energy absorption capacity. At the highest strain rate, the *η*_d_ of the specimen with *D*_f_ = 2.41 is approximately 37.1% and 16.4% higher than those of the specimens with *D*_f_ = 2.20 and *D*_f_ = 2.79, respectively.

This is mainly because a reasonable gradation can form a continuous and stable load-bearing skeleton, while fine particles effectively fill pores and enhance interfacial bonding. As a result, the specimen can absorb and dissipate more external input energy before failure. In contrast, when the fractal dimension is too low or too high, internal pore defects increase or the skeleton-supporting effect is weakened, making cracks more likely to propagate rapidly and reducing the effective energy dissipation capacity. This trend is consistent with the results of dynamic strength, DIF, and dynamic deformation modulus.

### 3.4. Macro- and Microscopic Failure Characteristics

**Figure 14:**
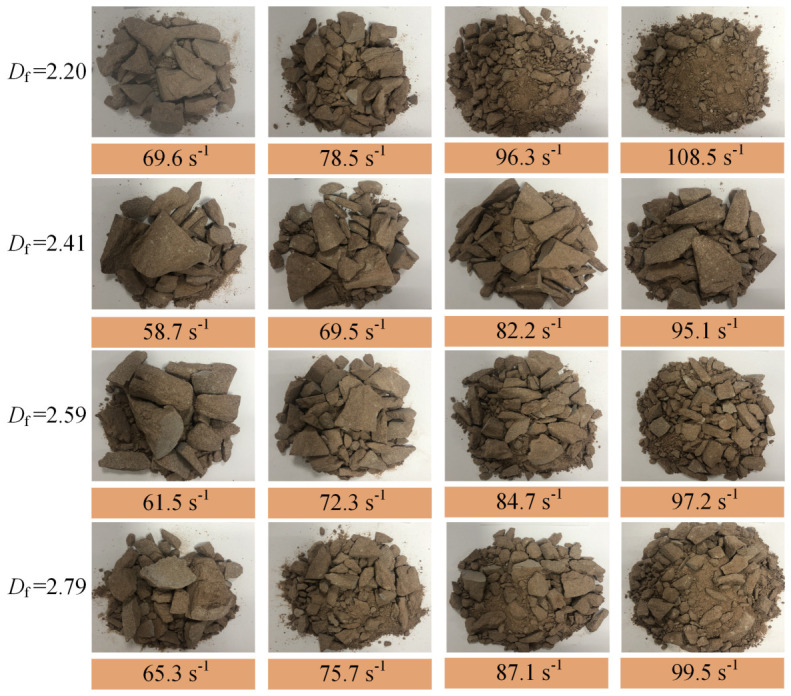
Dynamic macroscopic failure morphology of specimens.

The macroscopic failure morphology of specimens under impact loading can directly reflect their dynamic bearing capacity and energy dissipation characteristics. To analyze the effects of strain rate and aggregate fractal dimension on the failure mode of cemented coal gangue backfill composites, the fractured specimens were collected and observed after testing, as shown in Figure 14.

As shown in Figure 14, the failure degree of all specimen groups gradually intensifies with increasing strain rate. The failure morphology changes from blocky and flaky fragments at low strain rates to more pronounced fragmentation with increasing fine particles and powder at high strain rates. This indicates that, as the impact input energy increases, crack initiation, propagation, and coalescence inside the specimens become more sufficient, which is consistent with the continuous increase in incident energy and dissipated energy discussed above.

The fractal dimension also has a significant influence on the failure characteristics. At comparable strain-rate levels, the specimen with *D*_f_ = 2.41 retains more large fragments and contains fewer fine debris, indicating that its aggregate gradation is more reasonable and can form a continuous and stable load-bearing skeleton. In contrast, the specimen with *D*_f_ = 2.20 shows more fine fragments and powder at high strain rates, suggesting more severe failure due to discontinuous particle contacts and more developed pores and interfacial defects. The failure degree of the specimens with *D*_f_ = 2.59 and *D*_f_ = 2.79 lies between these two groups, while the latter shows a more evident pulverization tendency at high strain rates. Therefore, an intermediate fractal dimension is beneficial for improving the structural stability of the cemented composite and suppressing impact-induced fragmentation.

**Figure 15:**
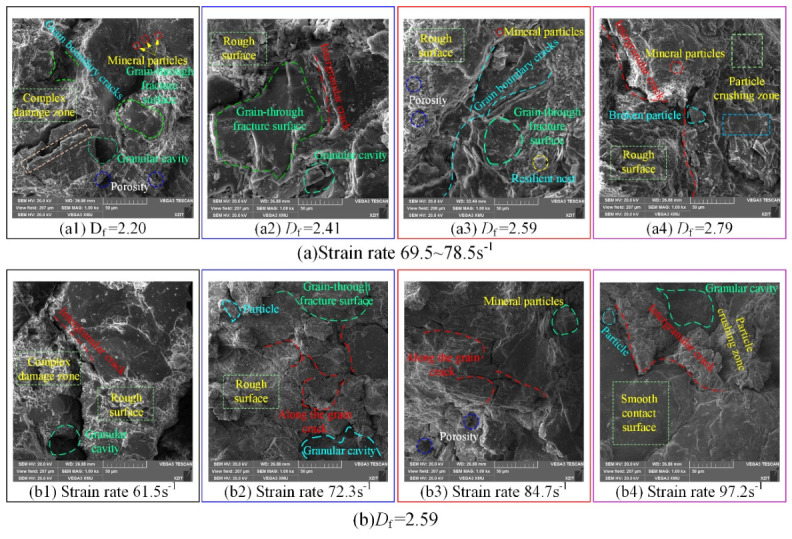
SEM micrographs of fractured surfaces after dynamic loading: (**a1**–**a4**) specimens with different fractal dimensions at comparable strain rates, including (**a1**) *D*_f_ = 2.20, (**a2**) *D*_f_ = 2.41, (**a3**) *D*_f_ = 2.59, and (**a4**) *D*_f_ = 2.79; (**b1**–**b4**) *D*_f_ = 2.59 specimens under different strain rates, including (**b1**) 61.5 s^−1^, (**b2**) 72.3 s^−1^, (**b3**) 84.7 s^−1^, and (**b4**) 97.2 s^−1^. The scale bar is indicated in each micrograph.

To further reveal the dynamic failure mechanism of cemented coal gangue backfill composites under different aggregate fractal dimensions and strain rates, scanning electron microscopy (SEM) was used to observe the fracture surfaces after impact failure, as shown in Figure 15. Figure 15 is organized into two comparison groups. The first group compares specimens with different fractal dimensions at comparable strain rates, whereas the second group compares the *D*_f_ = 2.59 specimens under different strain rates. The scale bar is indicated in each micrograph. To improve readability and avoid obscuring important microstructural features, only essential arrows and short labels are retained to indicate representative features, including pores, interfacial cracks, grain-through fracture surfaces, rough fracture surfaces, granular cavities, and local particle crushing zones. More detailed interpretations are provided in the main text. As shown in Figure 15a, under low strain-rate conditions, the fractal dimension has a clear influence on the fracture morphology. For the specimen with *D*_f_ = 2.20, many pores, particle pull-out pits, and complex damaged zones can be observed on the fracture surface, together with local rough interfaces and particle pull-out traces. This indicates that, at a low fractal dimension, the proportion of coarse particles is high, aggregate distribution is relatively discrete, and the interfacial transition zones are weaker. Under impact loading, cracks tend to propagate along the aggregate–paste interface and induce particle debonding.

As the fractal dimension increases to *D*_f_ = 2.41, the fracture surface becomes denser, and the particle interlocking effect is enhanced. More transgranular fracture surfaces and particle-boundary cracks can be observed, indicating that the coarse and fine particles are more reasonably graded under this condition. A continuous and stable load-bearing skeleton is formed inside the specimen, allowing stress to be effectively transferred into the aggregates. Consequently, the crack propagation mode changes from simple interfacial failure to the coexistence of interfacial failure and transgranular failure, which is consistent with the higher dynamic strength and dynamic deformation modulus of this group. When the fractal dimension further increases to *D*_f_ = 2.79, obvious particle crushing zones, broken particles, and local smooth contact surfaces appear on the fracture surface. This suggests that excessive fine particles weaken the skeleton-supporting effect, causing the impact load to be concentrated more in the cementitious matrix and local contact regions, thereby inducing particle crushing and brittle matrix failure.

It should be noted that the SEM observations provide qualitative evidence of local fracture-surface features, including pores, interfacial cracks, rough fracture surfaces, granular cavities, and local particle crushing. Therefore, the micrographs alone cannot fully quantify stress transfer or crack-deflection processes. In this study, interpretations related to crack-path tortuosity, interfacial bonding, and stress-transfer continuity are discussed as inferred mechanisms by combining SEM observations with the dynamic mechanical parameters, energy dissipation results, and macroscopic failure morphology. Accordingly, the SEM discussion focuses on observable features, and the mechanism-related statements are expressed in a more cautious manner.

Under the same fractal dimension condition, as shown in Figure 15b, increasing the strain rate significantly changes the crack propagation mode. At low strain rates, the fracture surface is dominated by relatively intact blocks and local interfacial cracks, with only a small number of intergranular cracks and pore defects, indicating a relatively simple crack propagation path and limited failure degree. As the strain rate increases, more intergranular cracks, transgranular fracture surfaces, and particle crushing traces can be observed. The fracture surface becomes rougher, and crack branching and intersection become more frequent.

When the strain rate reaches a high level, local particle crushing zones and continuous crack networks are formed, indicating that a large number of cracks initiate and coalesce within an extremely short time as the impact input energy increases rapidly. The failure mode gradually changes from simple tensile splitting to combined tensile–shear failure. This is consistent with the significant increase in dissipated energy and volumetric dissipated energy discussed above, meaning that more input energy is consumed by crack propagation, interfacial friction, and particle crushing.

Therefore, aggregate fractal dimension mainly affects crack initiation sites and propagation paths by regulating particle gradation, pore structure, and interfacial bonding state, whereas strain rate mainly controls crack propagation rate and failure intensity. Specimens with an intermediate fractal dimension have a denser internal structure and a more stable load-bearing skeleton, which can delay crack coalescence and absorb more energy under impact loading. When the fractal dimension is too low or too high, interfacial defects increase or the skeleton effect weakens, making cracks more likely to propagate rapidly and resulting in a decline in dynamic bearing capacity.

## 4. Dynamic Damage Mechanism Regulated by Fractal Gradation

Based on the static mechanical response, SHPB dynamic parameters, energy dissipation characteristics, and macro- and microscopic failure observations, the dynamic damage mechanism of cemented coal gangue backfill composites can be interpreted as a coupled process controlled by particle skeleton structure, stress-wave propagation, energy dissipation, and crack evolution. Because XRD or equivalent mineralogical characterization was not conducted in this study, the following discussion does not infer the contribution of specific mineral phases. Instead, the mechanism is discussed based on the experimentally supported features, including aggregate gradation, particle packing, pore defects, aggregate–matrix interfacial regions, stress-wave response, energy dissipation, and observed failure characteristics.

Fractal gradation determines the initial packing state and load-transfer skeleton of coal gangue aggregates, while strain rate controls the intensity and time scale of dynamic damage development. Therefore, the effect of fractal gradation under impact loading is not a simple particle-size effect, but a structural regulation process involving “particle skeleton structure–stress-wave propagation–energy dissipation–crack propagation.”

### 4.1. Effect of Fractal Gradation on Particle Skeleton and Initial Defects

Fractal gradation first affects the initial internal structure of cemented coal gangue backfill composites by changing the relative proportions of coarse and fine particles. These changes further control particle packing density, contact continuity, pore distribution, and the aggregate–matrix interfacial state. As a result, specimens with different fractal dimensions exhibit different initial defect structures and load-transfer paths before impact loading.

At a low fractal dimension, the aggregate system is dominated by coarse particles, whereas the filling effect of fine particles is insufficient. Although coarse particles can provide local skeleton support, the lack of fine-particle filling makes it difficult to form a continuous and stable contact network. Under this condition, larger pores, discontinuous contacts, and weak aggregate–matrix interfacial regions are more likely to exist inside the specimen. These initial defects can act as stress concentration zones during loading and provide preferential sites for crack initiation. Therefore, low-fractal-dimension specimens tend to show lower static strength, lower dynamic peak strength, and weaker deformation resistance.

At an intermediate fractal dimension, the coarse and fine particles form a more coordinated filling structure. Coarse particles provide the main load-bearing framework, while fine particles fill the pores between coarse aggregates and improve the compactness of the cemented system. This structure improves the continuity of the particle skeleton and enhances the contact state between aggregates and the cementitious matrix. Consequently, the external load can be transferred more uniformly through the aggregate skeleton and matrix, which helps improve both static bearing capacity and dynamic deformation resistance.

When the fractal dimension becomes too high, the proportion of fine particles increases excessively. Although fine particles can further fill local pores, excessive fine-particle content weakens the direct contact and mechanical interlocking among coarse particles. Under a fixed binder content, fine-particle-rich regions may also increase the dependence of the composite on the cementitious matrix for load bearing. As a result, the load-transfer path becomes less skeleton-dominated and more matrix-dominated. Such a structure is more prone to local compaction, matrix cracking, and fine-particle-related damage under impact loading. Therefore, excessive fine-particle filling is not necessarily beneficial for improving dynamic performance.

### 4.2. Coupling Between Stress-Wave Propagation and Energy Dissipation

During SHPB dynamic compression, the incident stress wave enters the specimen and propagates through a heterogeneous medium consisting of coal gangue aggregates, cementitious matrix, pores, and aggregate–matrix interfacial regions. The stability of stress-wave propagation is closely related to the continuity of the particle skeleton and the uniformity of the internal structure. A denser structure with better skeleton continuity can reduce local impedance mismatch and facilitate more stable stress transfer. In contrast, discontinuous particle contacts, pores, and weak interfaces can intensify wave scattering, reflection, and local stress concentration.

For low-fractal-dimension specimens, insufficient fine-particle filling results in a discontinuous coarse-particle skeleton and more developed initial defects. When the stress wave propagates through such a structure, local stress concentrations are more likely to form around pores and weak aggregate–matrix interfaces. Cracks may initiate early and propagate rapidly along these weak regions. Because failure occurs before the specimen fully mobilizes its energy-dissipation capacity, the dynamic bearing capacity and volumetric dissipated energy are relatively low.

For specimens with an intermediate fractal dimension, the more continuous coarse–fine particle skeleton provides a more stable path for stress-wave transmission. The improved contact continuity and denser structure help distribute impact stress more uniformly. Meanwhile, the more tortuous crack path and stronger aggregate–matrix interaction allow more impact energy to be consumed through crack initiation, crack branching, interfacial friction, matrix cracking, and local particle damage before final failure. This explains why specimens with intermediate fractal gradation generally exhibit higher dynamic strength, dynamic deformation modulus, and energy dissipation capacity among the tested gradations.

For high-fractal-dimension specimens, excessive fine particles weaken the coarse-particle skeleton and increase the role of matrix-dominated deformation and damage. Under impact loading, stress-wave propagation is more likely to induce local crushing or cracking in fine-particle-rich regions and cementitious matrix zones. Although these local damage processes may consume part of the input energy, they also accelerate damage accumulation and reduce the effective load-bearing capacity of the specimen. Therefore, the energy dissipation capacity of the composite is controlled not only by the magnitude of input energy, but also by whether the internal gradation structure can provide a stable skeleton and a distributed damage path.

### 4.3. Crack Evolution and Failure Mechanism Under Different Fractal Gradations

The final failure mode of cemented coal gangue backfill composites is governed by the coupling between stress-wave-induced damage, energy dissipation, and crack evolution. Under impact loading, cracks initiate preferentially at pores, weak aggregate–matrix interfacial regions, matrix defects, and local stress concentration regions. As the strain rate increases, the loading duration becomes shorter and the input energy increases, causing cracks to propagate and coalesce more rapidly. Therefore, the failure mode gradually evolves from relatively simple blocky fracture to more severe fragmentation and local pulverization.

For low-fractal-dimension specimens, crack propagation is mainly controlled by pores and weak aggregate–matrix interfaces caused by insufficient fine-particle filling. Once cracks initiate at these weak regions, they can rapidly connect along discontinuous contacts and interfacial defects. This leads to a relatively direct crack path and early loss of load-bearing capacity. Therefore, the failure of low-fractal-dimension specimens is more likely to be dominated by interfacial debonding and rapid crack coalescence.

For intermediate-fractal-dimension specimens, the coordinated coarse–fine particle structure improves skeleton continuity and compactness. Crack propagation is less likely to follow a single weak interface directly. Instead, cracks may be forced to bypass aggregates, branch through the cementitious matrix, or interact with local particle contacts. This more tortuous crack path increases energy consumption during failure and delays unstable crack coalescence. As a result, the specimens can maintain a higher dynamic bearing capacity and absorb more impact energy before final failure.

For high-fractal-dimension specimens, excessive fine-particle content weakens the coarse-particle skeleton and increases the susceptibility of fine-particle-rich matrix regions to local crushing and cracking. Under high strain-rate loading, cracks in these regions may propagate rapidly and interconnect, resulting in more severe matrix-dominated damage and local pulverization. This indicates that increasing the fine-particle proportion beyond a suitable range does not continuously improve impact resistance.

Overall, fractal gradation regulates the dynamic damage mechanism by controlling the initial particle skeleton, pore structure, aggregate–matrix interfacial state, stress-wave propagation path, and energy-dissipation mode. Among the four tested gradation conditions, the Df = 2.41 specimens show a more favorable balance between coarse-particle skeleton support and fine-particle filling, which contributes to improved dynamic bearing capacity, energy dissipation capacity, and impact stability. Therefore, the dynamic failure mechanism of cemented coal gangue backfill composites should be understood as a coupled skeleton–stress-wave–energy–crack evolution process rather than a monotonic effect of particle size alone.

## 5. Conclusions

In this study, different coal gangue aggregate gradations were designed based on mass fractal theory, and SHPB dynamic compression tests were conducted on cemented coal gangue backfill composites. By combining static mechanical parameters, dynamic strength, DIF, dynamic deformation modulus, energy evolution, and macro- and microscopic failure characteristics, the influence of aggregate fractal dimension on the dynamic mechanical response and damage failure mechanism of the material was systematically analyzed. The main conclusions are as follows:

(1) Cemented coal gangue backfill composites exhibit a pronounced strain-rate strengthening effect. With increasing strain rate, the dynamic peak strength, DIF, and dynamic deformation modulus all increase, and the dynamic peak strength shows a good correlation with strain rate. This indicates that under impact loading, crack initiation and propagation are constrained by the limited loading time, allowing the material to withstand higher instantaneous loads within a short period and thus exhibit stronger dynamic bearing capacity.
(2) Aggregate fractal dimension has a significant regulatory effect on the dynamic mechanical properties of the material. With increasing fractal dimension, the static strength, dynamic peak strength, DIF, and dynamic deformation modulus of the specimens generally first increase and then decrease. Among the tested groups, the specimen with *D*_f_ = 2.41 exhibits the best overall performance, with a maximum dynamic peak strength of 42.28 MPa and a maximum DIF of 3.12. This indicates that a reasonable fractal gradation promotes synergistic filling between coarse and fine particles and forms a continuous and stable load-bearing skeleton, thereby improving the dynamic strength and deformation resistance of the material.
(3) The energy evolution results show that the incident energy, reflected energy, transmitted energy, and dissipated energy all increase with increasing strain rate, among which the dissipated energy and volumetric dissipated energy are more sensitive to changes in fractal dimension. Specimens with intermediate fractal dimensions exhibit higher energy absorption and dissipation capacities, indicating that a reasonable gradation not only facilitates stable stress wave propagation but also enables the specimens to consume more energy through crack propagation, interfacial friction, and particle crushing before impact failure, thereby improving impact stability.
(4) The macro- and microscopic failure results indicate that, with increasing strain rate, the failure mode of the specimens changes from blocky fracture to fragmentation and pulverization. When the fractal dimension is low, the proportion of coarse particles is high, and interfacial defects and pore structures are more developed; thus, cracks tend to propagate rapidly along the aggregate–paste interface. When the fractal dimension is high, the proportion of fine particles is excessive, and the supporting effect of the coarse-particle skeleton is weakened, making matrix crushing and particle breakage more likely to occur. In contrast, specimens with an intermediate fractal dimension have a denser internal structure and more tortuous crack propagation paths, with interfacial failure and transgranular failure developing cooperatively, thereby showing superior resistance to dynamic damage.

## Figures and Tables

**Table 1:** Major oxide compositions of raw materials (wt.%).

Materials	SiO_2_	Al_2_O_3_	CaO	Fe_2_O_3_	MgO	Na_2_O	K_2_O
Cement	21.60	9.58	54.31	3.25	2.10	0.30	0.65
Fly ash	52.19	26.49	6.12	7.18	1.87	0.89	2.11
Gangue	58.24	26.45	1.81	4.27	1.42	0.66	3.15

## Data Availability

The original contributions presented in this study are included in the article. Further inquiries can be directed to the corresponding author.
